# Blockage of Eosinopoiesis by IL-17A Is Prevented by Cytokine and Lipid Mediators of Allergic Inflammation

**DOI:** 10.1155/2015/968932

**Published:** 2015-06-23

**Authors:** Pedro Xavier-Elsas, Bianca de Luca, Túlio Queto, Bruno Marques Vieira, Daniela Masid-de-Brito, Elizabeth Chen Dahab, José Carlos Alves Filho, Fernando Q. Cunha, Maria Ignez C. Gaspar-Elsas

**Affiliations:** ^1^Department of Immunology, Paulo de Góes Institute for Microbiology, Federal University of Rio de Janeiro (UFRJ), 21941-590 Rio de Janeiro, RJ, Brazil; ^2^Department of Pediatrics, Fernandes Figueira National Institute for Health of Women, Children and Adolescents, Oswaldo Cruz Foundation (FIOCRUZ), 22250-020 Rio de Janeiro, RJ, Brazil; ^3^Department of Pharmacology, Medical School of Ribeirão Preto, University of São Paulo, 14049-900 Ribeirão Preto, SP, Brazil

## Abstract

Interleukin- (IL-) 17A, a pleiotropic mediator of inflammation and autoimmunity, potently stimulates bone-marrow neutrophil production. To explore IL-17A effects on eosinopoiesis, we cultured bone-marrow from wild-type mice, or mutants lacking inducible nitric oxide synthase (iNOS−/−), CD95 (*lpr*), IL-17RA, or IL-4, with IL-5, alone or associated with IL-17A. Synergisms between IL-17A-activated, NO-dependent, and NO-independent mechanisms and antagonisms between IL-17A and proallergic factors were further examined. While IL-17A (0.1–10 ng/mL) had no IL-5-independent effect on eosinopoiesis, it dose-dependently suppressed IL-5-induced eosinophil differentiation, by acting during the initial 24 hours. Its effectiveness was abolished by caspase inhibitor, zVAD-fmk. The effect of IL-17A (0.1–1 ng/mL) was sensitive to the iNOS-selective inhibitor aminoguanidine and undetectable in iNOS−/− bone-marrow. By contrast, a higher IL-17A concentration (10 ng/mL) retained significant suppressive effect in both conditions, unmasking a high-end iNOS-independent mechanism. Lower IL-17A concentrations synergized with NO donor nitroprusside. Eosinopoiesis suppression by IL-17A was (a) undetectable in bone-marrow lacking IL-17RA or CD95 and (b) actively prevented by LTD4, LTC4, IL-13, and eotaxin. Sensitivity to IL-17A was increased in bone-marrow lacking IL-4; adding IL-4 to the cultures restored IL-5 responses to control levels. Therefore, effects of both IL-17A and proallergic factors are transduced by the iNOS-CD95 pathway in isolated bone-marrow.

## 1. Introduction

The initial description of interleukin- (IL-) 17 and its receptor, almost two decades ago [1], generated great interest in the properties and interactions of this cytokine and of related molecules, as well as on the novel profiles of cells that produce them. IL-17A is the prototype of a family of 6 structurally related mammalian cytokines [2–5], with affinity for specialized receptors which themselves belong to another novel gene family [6, 7]. IL-17A plays important roles both in host defense against various classes of infectious pathogens [8–10] and in the immunological dysfunctions that underlie conditions as challenging as organ-specific autoimmunity [11], autoimmune/rheumatoid arthritis [12, 13], sepsis [14], cancer [15], and atopic dermatitis [11, 16]. The expression of IL-17 family cytokines is a defining feature of CD4+ TH17 lymphocytes and other lymphoid cell subsets [6, 17] and a marker/inducer of activation for epithelial cells in inflammatory diseases of skin [18], lung [19], intestine [20], and other sites. In addition, previous hematological studies highlighted a strong in vivo stimulatory effect of IL-17A on the neutrophil lineage [21, 22], which is central to immunity against bacteria and fungi [23]. IL-17A regulates granulocyte populations by coupling granulocyte clearance from tissues to neutropoiesis in bone-marrow through induction of the granulocyte colony-stimulating factor (G-CSF) [24]. By contrast, less is known about IL-17A effects on eosinophils, which are closely related to neutrophils [25] but remain better known for their pathogenetic roles in allergy than for their contributions to antimicrobial defences [26].

In chronic allergic reactions, IL-17 family cytokines are associated with the more destructive manifestations, which involve contributions from T lymphocytes, neutrophils, eosinophils, and mast cells [27]. Nevertheless, the relationship between IL-17A and the cytokine environment of allergy is complex. In humans, TH17-selective expression of IL-13 receptor *α* chain allows IL-13 to suppress IL-17A production [28]. IL-17A production is also inhibited by IL-4 [29]. In a murine asthma model, IL-17 promotes neutrophilia while inhibiting eosinophilia through central (bone-marrow) and peripheral (lung) actions [29]. Furthermore, in mice lacking both IL-4 and IL-13, IL-17 overproduction induced by allergic sensitization elicits a neutrophilic inflammatory infiltrate in allergen-challenged airways, with scant eosinophils [30]. Finally, G-CSF, the neutropoietic cytokine induced by IL-23 via IL-17A [24], suppresses eosinopoiesis in vivo and ex vivo, in a murine asthma model [31]. These studies suggest that IL-17A favors the production of neutrophils over eosinophils, thereby contributing to restricting the eosinophilia of allergic reactions. Nevertheless, these in vivo studies do not distinguish between possible* direct* effects of IL-17A on eosinophil precursors and* indirect* effects mediated by other factors, like IL-23 and G-CSF [24], not necessarily generated within the hemopoietic compartment.

Murine eosinopoiesis is suppressed by a proapoptotic pathway requiring the inducible isoform of nitric oxide (NO) synthase (iNOS) and the ligand for death receptor CD95 (CD95L) [32]. This pathway, activated in bone-marrow culture by prostaglandin E_2_ (PGE_2_), also mediates the in vivo suppression of eosinophilia in bone-marrow and lungs of sensitized and challenged mice by the leukotriene synthesis inhibitor, diethylcarbamazine (DEC) [33]. The suppressive effect of PGE_2_ is blocked by the cysteinyl-leukotrienes (CysLT, comprising LTC_4_, LTD_4_, and LTE_4_) [34]. The CysLT are produced through the 5-LO pathway, which is absolutely required for the in vivo effectiveness of DEC [35]. CysLT also enhance eosinopoiesis in the absence of PGE_2_ [34] and mediate the eosinopoietic effects of both eotaxin and IL-13 in bone-marrow culture [36]. Hence, the iNOS-CD95L pathway may play two distinct roles, by (a)* directly* transducing the proapoptotic effects of soluble activators (PGE_2_ and other agonists which signal through cAMP [37]) and (b)* indirectly* conveying the antiapoptotic effects of proallergic cytokine and lipid mediators (CysLT, eotaxin, and IL-13). Here we examined the effects of IL-17A on eosinopoiesis and the roles of iNOS/CD95, caspases [37], and IL-17RA therein, as well as the outcome of IL-17A interactions with cytokine and lipid proallergic mediators.

## 2. Methods

### 2.1. Animals and Animal Procedures

Male and female BALB/c mice, wild-type ones, and mutants lacking either IL-4 (IL-4 KO; Jackson Laboratory online reference http://jaxmice.jax.org/strain/002496.html) functional genes or an enhancer element in the promoter region of gene coding for the GATA-1 transcription factor [26], required for eosinophil-lineage determination (GATA-1 dbl KO or GATA-1 for short; Jackson Laboratory online reference http://jaxmice.jax.org/strain/005653.html), and C57BL/6 mice, wild-type ones, and mutants lacking iNOS [33, Jackson reference #002596] functional genes were bred at CECAL-FIOCRUZ (Rio de Janeiro, Brazil) and used at 6–8 weeks of age. Animal housing and handling followed procedures approved by the Institutional Committee on Ethical Handling of Laboratory Animals (Licenses CEUA #L002-09; CEUA-CCS-UFRJ #181-13). Mutant mice lacking functional IL17RA [39] or CD95 genes (B6.MRL-Faslpr/J; Jackson Laboratory online reference http://jaxmice.jax.org/strain/000482.html), bred at the Department of Pharmacology, Ribeirão Preto Medical School, University of São Paulo, Brazil, along with the respective wild-type controls, were also used as approved by the Institutional Ethics Committee (CEUA-CCS-UFRJ #181-13).

### 2.2. Reagents

FCS and culture media were from Hyclone (Logan, UT, USA); aminoguanidine, Agar Noble, L-glutamine, sodium nitroprusside (SNP), zVAD-fmk, dexamethasone, and polymyxin E (colistin) sulfate were from Sigma Chemical Co. (St. Louis, MO, USA); penicillin-streptomycin solution was from GIBCO (Life Technologies, São Paulo, SP, Brazil); LTC_4_, LTD_4_ were from Cayman Chemical Co. (Ann Arbor, MI, USA); recombinant murine (rm) IL-17A, GM-CSF, IL-5, IL-13, and eotaxin were from R&D Systems (Minneapolis, MN, USA); rmIL-4 was from Peprotech (Rocky Hill, NJ); liquid diaminobenzidine solution was from Dako Cytomation (Dako Denmark A/S, Glostrup, Denmark).

### 2.3. Bone-Marrow Cell Studies

Bone-marrow cells were obtained by flushing the two femurs of each mouse with RPMI-1640 medium containing 1% FCS, followed by washing, resuspension in medium with 10% FCS, staining, and total and differential counts. These were carried out in a haemocytometer and on cytocentrifuge slides, respectively, stained for nucleated cells with crystal violet and for eosinophil peroxidase- (EPO-) positive cells with DAB in the presence of KCN 6 mM [40]. EPO is an eosinophil-selective marker, detectable at all stages, from the earliest precursors to terminally differentiated cells in inflammatory sites [40]. Throughout this study, to assess the effects of different interventions, liquid culture assays were used, in order to facilitate sequential addition and proper mixing of the reagents to previously plated cells. Liquid cultures were established by seeding 10^6^ bone-marrow cells from individual mice in 1 mL RPMI-1640 medium, with 10% FCS in the presence of IL-5 (1 ng/mL; alternatively, GM-CSF 1 ng/mL, where indicated), alone or in association with IL-17A (0.1–10 ng/mL) and incubated at 37°C, 5% CO_2_/95% air, for 7 days, before collecting and submitting the cells to total and differential counts as above. These conditions were previously shown to support eosinophil proliferation and terminal differentiation and to allow detection of enhancing and suppressive effects [31–37]. As a rule, inhibitors (zVAD-fmk, aminoguanidine, and polymyxin) were added before positive stimuli (IL-5, GM-CSF), cytokine, or lipid mediators (IL-17, IL-4, IL-13, and eotaxin or LTC_4_, LTD_4_) or NO-generating chemicals (SNP) [32], all being present from the beginning of the culture without replenishment, unless otherwise specified. In selected experiments, the ability of IL-17 to interact with dexamethasone, inducing terminal maturation rather than apoptosis, was examined following published protocols [38]. Where indicated, additional experiments to define whether IL-17A had an impact on lineage-committed progenitors (colony-forming cells) were carried out in semisolid (clonal) cultures [36], established from 2 × 10^5^ cells in 1 mL, in 35 mm triplicate culture dishes, in a mixture of IMDM with 20% FCS and agar Noble (0.3% final concentration), containing GM-CSF (2 ng/mL), alone or in association with IL-17A (0.1–10 ng/mL). Colonies (>50 cells) were scored at day 7. Colonies were subsequently typed in agar layers which had been dried, mounted on microscope slides, stained for EPO, and scored under high magnification (400x). Where indicated, bone-marrow progenitor numbers were expanded in liquid culture with Flt3L and Stem Cell Factor, before induction of eosinopoiesis by a mixture of cytokines (GM-CSF, IL-3, and IL-5), also in liquid culture, according to Dyer et al. [41]. Where indicated, cells from 2-day or 5-day cultures established with BALB/c or GATA-1 bone-marrow were harvested, washed in PBS, and stained with Annexin V-FITC and propidium iodide (PI) using a TACS Annexin V Kit from Trevigen (Gaithersburg, MD; Catalog # 4830-01-K), according to the manufacturers' instructions. Flow cytometry was carried out in a FACScalibur after gating in the granulocyte region and excluding PI-stained (necrotic) cells on FL-2. Annexin V-FITC-stained cells (positive in FL-1) in this gate were acquired up to 20.000 events per sample. Data were analysed with Flowing software 2.

### 2.4. Statistical Analysis

Data (mean + SEM) were analyzed with Systat for Windows, Version 4, from Systat Inc. (Evanston, IL, USA), using factorial ANOVA with the Tukey (HSD) correction for multiple comparisons of means observed with different treatments or the two-tailed *t*-test for comparisons of two groups.

## 3. Results

### 3.1. Characterization of Effects of IL-17A on Eosinophil Production

**Figure 1:**
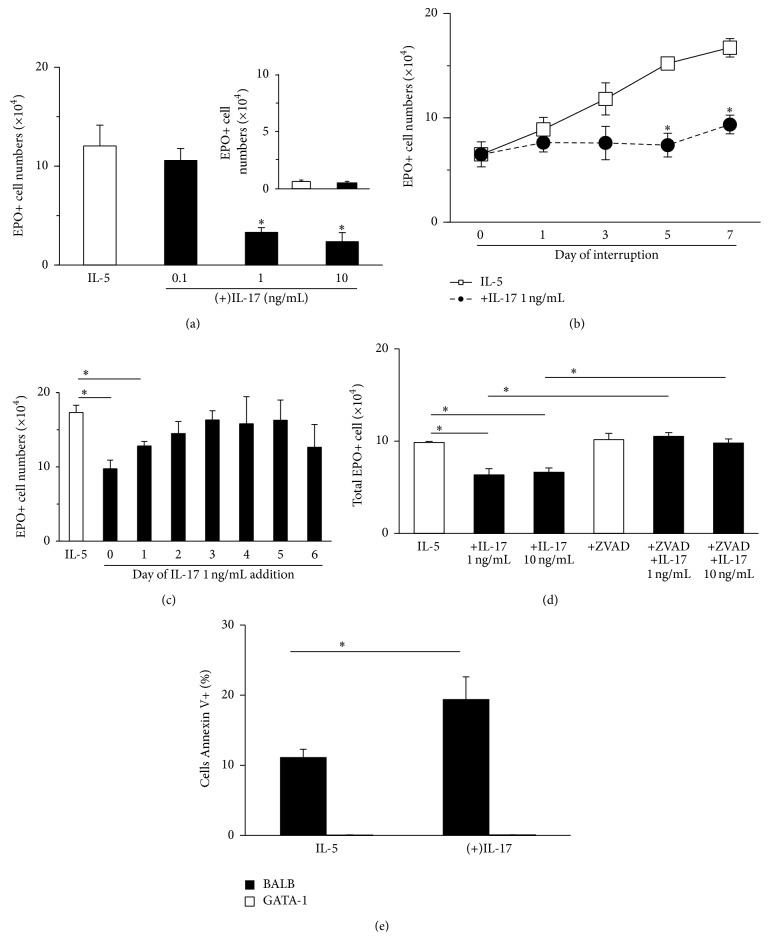
Effect of IL-17A on eosinophil production from murine bone-marrow. Bone-marrow cultures were established from BALB/c mice in the presence of IL-5 (1 ng/mL), alone or in association with the indicated concentrations of IL-17A. After the indicated periods, cells were collected, counted, and stained for eosinophil peroxidase (EPO). Total and EPO+ cell counts were performed, and data (mean ± SEM, *n* = 4) are the numbers of EPO+ cells present at the end of the culture. (a) Effect of varying IL-17A concentration on EPO+ cell numbers at day 7.* Insert*: effect of culture in the absence of IL-5; open and closed bars: cultures established, respectively, with and without IL-17A. (b) Effect of increasing duration of culture. (c) Effect of variable timing of IL-17A addition relative to the initiation of the culture. (d) Effect of blocking terminal caspases with zVAD-fmk (z-VAD) (20 *μ*M). (e) Annexin V-FITC staining of cells in the granulocyte gate recovered from BALB/c and GATA-1 day 5 bone-marrow cultures. Data are mean ± SEM (*n* = 3) of the % granulocytes in the gated region (FL-1 positive, FL-2 negative). At day 2, there was no significant difference between IL-17A-exposed and control cultures from BALB/c mice (not shown). ^*∗*^ *P* < 0.05 for the differences between the indicated points and the respective IL-5 (negative) controls.

Initially, we characterized the effects of IL-17A on IL-5-induced eosinopoiesis (Figure 1), regarding its concentration-response relationship, kinetics, and window of opportunity. We further evaluated whether IL-17A had an effect in the absence of IL-5 (Figure 1(a), insert) and whether its suppressive effects, as expected in apoptotic processes, depended on caspases and were associated with increased phosphatidylserine externalization. IL-17A addition to bone-marrow cultures established from naive BALB/c donors, for 7 days, in the presence of IL-5, concentration-dependently suppressed eosinopoiesis (Figure 1(a)), with significant effects at 1 and 10 ng/mL, but not 0.1 ng/mL. Importantly, IL-17A only had a detectable effect in the presence of IL-5 (Figure 1(a), insert). While IL-5 alone supported a progressive increase in the number of eosinophils recovered up to day 7 (Figure 1(b)), IL-17A prevented this increase, with a significant effect from day 5 onwards. This type of kinetics was similar to that previously described for PGE_2_, the prototypical activator of the iNOS-CD95L proapoptotic mechanism [30]: like PGE_2_ [38], IL-17A must act at the beginning of the culture (up to 24 h) and has no significant effect if it is introduced at later times (Figure 1(c)). This effect of IL-17A (1–10 ng/mL) is abolished by zVAD-fmk, showing its dependence on caspases (Figure 1(d)). Importantly, zVAD-fmk had no effect in the absence of IL-17A. Annexin V-binding cells in the granulocyte gate were found in significantly increased numbers (Figure 1(e)), in IL-17A-exposed cultures relative to IL-5 control cultures, by day 5, when an effect of IL-17 on eosinophil numbers becomes demonstrable (Figure 1(b)), but not by day 2 (not shown). Importantly, this increase was not observed in cultures from GATA-1 donors, which do not produce eosinophils in the presence of IL-5 but do present cells in the granulocyte gate, which survive from the bone-marrow inoculum. Hence, increased Annexin V-binding in day 5 cultures is correlated with the presence of eosinophil-lineage cells and with exposure to IL-17A during the initial 24 h.

**Figure 2:**
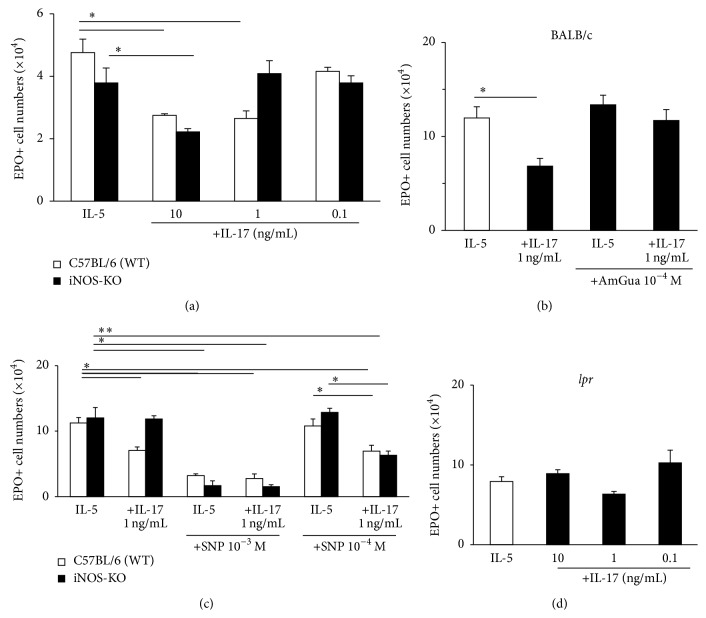
Dependence of IL-17A on iNOS and synergism between IL-17A and exogenous NO in iNOS-deficient bone-marrow. Bone-marrow cultures were established with IL-5, alone or in association with IL-17A, from wild-type (WT) control (C57BL/6, white bars) and iNOS-deficient (iNOS-KO, black bars) mutant C57BL/6 mice (a, c) or from BALB/c wild-type mice (b) or from CD95-deficient* lpr* mutant BALB/c (d), as described in legend of Figure 1. Data (mean ± SEM), *n* = 3 (a, c, and d), *n* = 4 (b), are the numbers of EPO+ cells recovered at day 7. (a) Concentration-response relationship for IL-17A on WT and iNOS-deficient bone-marrow. (b) Blockade of IL-17A effect by iNOS-selective inhibitor aminoguanidine (AmGua). (c) Concentration-response relationships for IL-17A and SNP, separately and in combination, in wild-type and iNOS-deficient bone-marrow, showing synergism between IL-17A (1 ng/mL) and NO donor SNP (10^−4^ M) in iNOS-deficient bone-marrow. (d) Dependence of IL-17A effectiveness on CD95 (comparing with (b), white bar and first black bar). ^*∗*^ *P* < 0.05 and ^*∗∗*^ *P* < 0.01 for the differences between the indicated points or in (b), relative to the respective IL-5 (negative) controls.

We further examined whether iNOS and CD95 were required for suppression of eosinopoiesis by IL-17A (Figure 2). IL-17A, 1 ng/mL, had no effect on bone-marrow from iNOS-deficient donors, in contrast to its ability to suppress eosinopoiesis in bone-marrow from B6 wild-type control donors (Figure 2(a)). Moreover, selective iNOS inhibitor aminoguanidine, 10^−4^ M, prevented the effect of IL-17A, 1 ng/mL (Figure 2(b)). In the absence of IL-17A, aminoguanidine had no significant effect. The effect of iNOS inactivation (Figure 2(a)) or inhibition (Figure 2(b)) was dependent on the concentration of IL-17A in the cultures: bone-marrow from both iNOS-deficient and wild-type donors remained sensitive to IL-17A, provided that it was present at a 10-fold higher concentration (Figure 2(a)); similarly, in BALB/c bone-marrow, aminoguanidine was unable to prevent the effects of IL-17A, 10 ng/mL (not shown). Therefore, only partial blockade of IL-17A effects was achieved by interference with iNOS, suggesting the existence of two mechanisms of suppression, one operative at a lower IL-17A concentration (1 ng/mL) and mediated by iNOS and another detectable at 10-fold higher concentration (10 ng/mL), which is iNOS-independent. We further examined the possibility of synergism between exogenously introduced NO and IL-17A in the absence of iNOS: in iNOS-deficient bone-marrow, IL-17A, 1 ng/mL (a concentration insufficient to suppress eosinopoiesis; see Figure 2(a)), synergized with SNP, 10^−4^ M (which is also insufficient to suppress eosinopoiesis; see Figure 2(c)), resulting in significant suppression. The ability of a 10-fold higher concentration of SNP (10^−3^ M) to suppress eosinopoiesis, even in the absence of iNOS, is consistent with previous observations [30] that NO donors override the requirement for functional iNOS. Finally, the effects of IL-17A were abolished in bone-marrow lacking CD95 (*lpr*), as expected from the involvement of the iNOS-CD95L proapoptotic pathway (Figure 2(d)).

The cumulative evidence from Figures 1 and 2 led us to hypothesize that IL-17A has very similar, if not identical, effects to those of PGE_2_ in the same experimental conditions [32, 34, 37], inducing apoptosis by a mechanism dependent on both iNOS and CD95. The similarities between IL-17A and PGE_2_ further extend to the promotion of eosinophil terminal differentiation [38], observed when either IL-17A or PGE_2_ is allowed to interact with dexamethasone, which prevents iNOS expression [37] (not shown).

**Figure 3:**
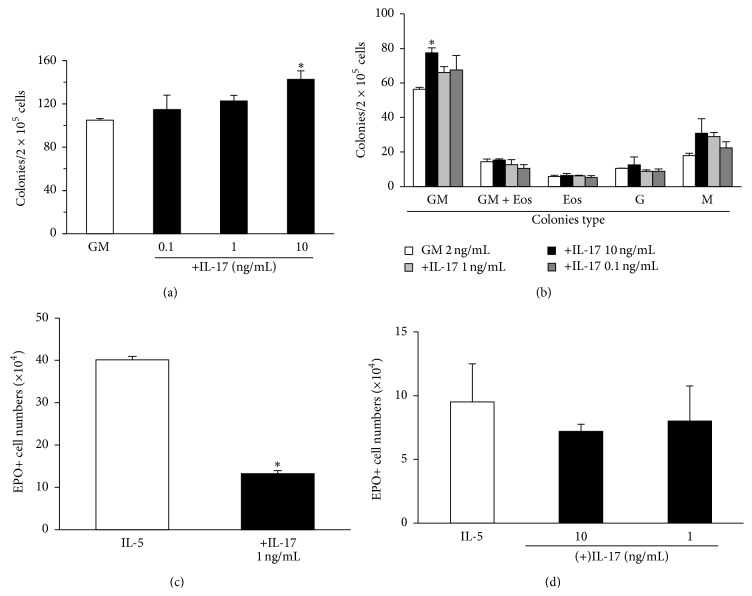
IL-17, effect on progenitor responses and requirement for IL-17RA. (a, b) Semisolid cultures were established for 7 days, from 2 × 10^5^ bone-marrow cells of naive BALB/c mice, in the presence of GM-CSF (2 ng/mL), alone or in association with IL-17A at the indicated concentrations. Total (a) and differential (b) colony counts were carried out under the inverted microscope, directly (a) or after staining dried agar layers for EPO and counterstaining by Harris' Hematoxylin (b). Colony types in (b) are [36] pure granulocyte-macrophage (GM), mixed granulocyte-macrophage-eosinophil (GMEos), pure eosinophil (Eos), pure granulocyte (G), and pure macrophage (M). GM-CSF alone, black bar. GM-CSF in association with IL-17A (0.1 to 1 ng/mL), white bars. (c) Liquid cultures were established as described by Gaspar-Elsas et al. [38], in a two-phase culture with progenitor expansion in Flt3L and SCF, followed by eosinophil differentiation for up to 10 days with GM-CSF, IL-3, and IL-5, in the absence (white bar) or presence (black bar) of IL-17A (1 ng/mL). (d) Liquid cultures were established as in Figure 1(a), with bone-marrow from BALB/c mutants lacking IL-17RA. Data are mean ± SEM. ^*∗*^ *P* < 0.05. Significant differences relative to GM controls (a, b) or IL-5 controls (c, d). All groups, *n* = 3.

Because PGE_2_ has a strong inhibitory effect on myeloid colony formation [42], while IL-17 is considered a strong stimulant of granulopoiesis in vivo [21, 22, 24], we examined in detail the effects of IL-17A on myeloid colony-forming cells (progenitors). We evaluated whether IL-17A significantly affected colony formation in the presence of GM-CSF (Figures 3(a) and 3(b)), taking care to distinguish between effects on total colony counts (Figure 3(a)) and on different colony types (Figure 3(b)). In the 0.1–1 ng/mL concentration range, IL-17A had no significant effect on colony counts, total or differential, in comparison with the GM-CSF control. By contrast, IL-17A had a significant enhancing effect on total colony counts at 10 ng/mL (Figure 3(a)). This effect was accounted for by a significant increase in granulocyte-macrophage (GM) colony counts (Figure 3(b)), but not in eosinophil-containing GM colonies (GMEos), which were scored as a separate category [36], nor in pure eosinophil colonies (Eos). All myeloid colony types other than pure GM colonies, including both classes of eosinophil-containing colonies, were unaffected by IL-A17 over the 0.1–10 ng/mL concentration range. Therefore, no suppression of eosinophil progenitors was observed in semisolid culture, suggesting that the inhibition of eosinopoiesis by IL-17A is due to an effect on lineage-committed precursors, which are but no longer able to form colonies.

The lack of suppression of eosinopoiesis by IL-17A in GM-CSF-stimulated colony formation assays (Figure 3(b)), as opposed to the suppression of eosinopoiesis by IL-17A in IL-5-stimulated liquid cultures (Figure 1), might be related to one of two mechanisms: (a) a protective effect of GM-CSF against the suppressive effect of IL-17A or (b) the inability of IL-17A to suppress eosinopoiesis in conditions of intense hemopoietic cell proliferation, as it is typically induced by GM-CSF, a more effective proliferative stimulus than IL-5. We therefore examined whether strong expansion of progenitor numbers by exposure to Flt3L and Stem Cell Factor, followed by change of medium and further culture in a cocktail of GM-CSF, IL-3, and IL-5 [42], would reduce the effectiveness of IL-17A in suppressing eosinopoiesis. As shown in Figure 3(c), this more complex culture protocol resulted in a significantly increased number of eosinophils recovered at the end of the culture, relative to cultures with IL-5 alone (comparing with Figure 1(a)), which is explained by the strong expansion of progenitors as well as by the synergic interaction of the eosinopoietic cytokines, GM-CSF, IL-3, and IL-5 [42]. Nevertheless, the yield of eosinophils was strikingly reduced if IL-17A was present during the eosinopoietic phase of the culture, along with the mixture of GM-CSF, IL-3, and IL-5. This shows that the absence of a suppressive effect of IL-17A in semisolid cultures established in the presence of GM-CSF is not due to any protective effect of GM-CSF against IL-17A suppressive actions. It also rules out the possibility that the strong proliferative response of hemopoietic cells to GM-CSF somehow blocks the ability of IL-17A to suppress bone-marrow eosinopoiesis.

Importantly, we confirmed that the suppressive effect of IL-17A in liquid culture required a functional IL-17RA [6, 7]. No significant effect of IL-17 was observed in cultures from IL-17RA KO mice (Figure 3(d); compare with Figure 1(a)). Also, polymyxin, an effective neutralizer for endotoxin [43], did not change the effectiveness of IL-17A 1–10 ng/mL, when present in excess relative to the expected level of residual endotoxin [44]. Together, these observations rule out the possibility of the effect of IL-17A 1 ng/mL being accounted for by carryover of LPS, rather than by the cytokine itself, a possibility raised by some studies [44].

### 3.2. Ability of Proallergic Mediators to Prevent the Suppressive Effects of IL-17A

Previous studies had established that IL-5-stimulated eosinopoiesis can be enhanced by the cysteinyl-leukotrienes (CysLT), LTC_4_, LTD_4_, and LTE_4_, which all share an ability to activate type I CysLT receptors (CysLT1R) [34]. CysLT were further shown to protect eosinopoiesis from the suppressive effects of PGE_2_ [34]. Importantly, pharmacological blockade or knocking out CysLT1R abolished both enhancing and protective activities of CysLT. In addition, indomethacin [34], IL-13, and eotaxin [36] were all shown to have enhancing and protective effects that were equally abolished by blockade or inactivation of CysLT1R.

**Figure 4:**
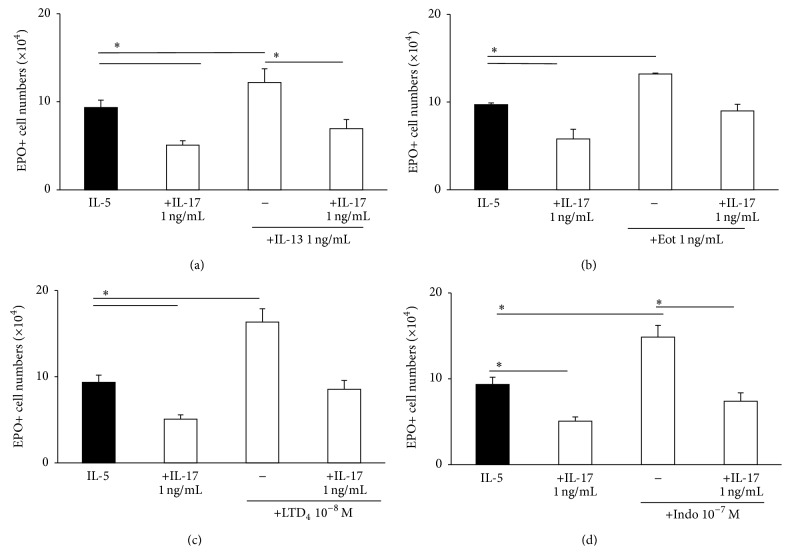
Blockade of IL-17 effects by agents which signal through CysLT1R. Liquid bone-marrow cultures were established from BALB/c mice as detailed in Figure 1. Different agonists which share the ability to signal through CysLT type 1 receptors (CysLT1R) were added to the cultures, alone or in association with IL-17A. These include (a) IL-13 and (b) eotaxin (Eot) [36] and (c) leukotriene D_4_ (LTD_4_) and (d) indomethacin [34]. ^*∗*^ *P* < 0.05 for the indicated differences. Data are mean ± SEM (*n* = 3).

Because the common biochemical mechanism shared by these heterogeneous agents was known to prevent suppression by PGE_2_, we further examined whether IL-13, eotaxin, LTD_4_, and indomethacin would be equally effective in blocking the actions of IL-17A (Figure 4). Consistently with this hypothesis, both IL-13 (Figure 4(a)) and eotaxin (Figure 4(b)) prevented suppression of eosinopoiesis by IL-17A, in addition to enhancing eosinopoiesis in the absence of IL-17A. LTD_4_, 10^−8 ^M, abolished the suppressive effect of IL-17A, 1 ng/mL (Figure 4(c)), in addition to enhancing eosinopoiesis in the absence of IL-17A. Similar observations were made with LTC_4_ (not shown). Finally (Figure 4(d)), indomethacin was able to enhance eosinopoiesis and to block the effect of IL-17A. In all cases, blockade by all four agents brought eosinophil production back to the IL-5 control levels.

**Figure 5:**
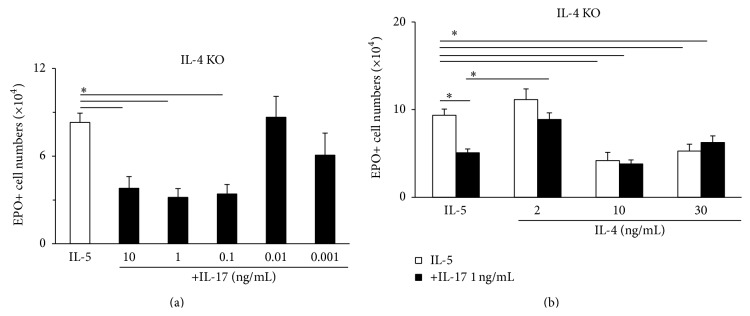
IL-4 attenuates the suppressive effects of IL-17A on eosinopoiesis. Bone-marrow cultures were established with IL-5, alone or in association with IL-17A, IL-4, or both, from IL-4-deficient mice of the BALB/c background mice, as described in legend of Figure 1. Data (mean + SEM) are the numbers of EPO+ cells recovered at day 7. (a) Concentration-response relationship for IL-17A with significant suppression down to 0.1 ng/mL (comparing with Figure 1(a)). (b) Effect of adding IL-4 in different concentrations to cultures in the absence (white bars) and in the presence (black bars) of IL-17, 1 ng/mL. 2 ng/mL abolished the suppressive response to IL-17A, which is highly effective in the absence of exogenous IL-4. Data are mean ± SEM of the numbers of EPO+ cells recovered at day 7. ^*∗*^ *P* < 0.05 for the indicated differences.

The ability of cytokine and lipid mediators of allergic inflammation, such as CysLT, eotaxin, and IL-13, to prevent IL-17A actions on eosinopoiesis, in a way consistent with the ability of all these agents to enhance IL-5-dependent eosinopoiesis, prompted us to evaluate whether the prototypical TH2 cytokine, IL-4, which shares signaling mechanisms with IL-13, would act similarly. As shown in Figure 5, bone-marrow from mice lacking IL-4 production had a 10-fold lower threshold for the inhibitory effect of IL-17A (Figure 5(a)) than bone-marrow from WT BALB/c controls (Figure 1(a)). This sensitivity increased by one order of magnitude suggested that IL-4 acts in vivo to attenuate responsiveness to IL-17A. If so, addition of IL-4 in vitro should be able to restore responses to IL-17A in IL-4-deficient bone-marrow back to the level of WT controls. This is indeed confirmed by the data in Figure 5(b): in IL-4-deficient bone-marrow, IL-4 at a relatively low concentration in culture (2 ng/mL) abolished the effectiveness of IL-17A, 1 ng/mL, which in the absence of IL-4 was highly effective. At a higher concentration range (10–30 ng/mL), IL-4 suppressed eosinopoiesis, in a way that was both independent of and unaffected by the presence of IL-17A and possibly represented an unrelated regulatory effect of IL-4.

## 4. Discussion

This is, to our knowledge, the first demonstration that IL-17A has a strong suppressive effect on eosinopoiesis by acting directly in bone-marrow culture. This opposes the view that IL-17A is generally stimulatory to granulopoiesis [21, 22, 24] and highlights the need for more detailed characterization of the lineage-selective effects of IL-17A. Nevertheless, it is consistent with a number of reports that IL-17A, either overexpressed in vivo through specific vectors [16] or produced endogenously as part of immune responses in wild-type [29] or mutant mice [30], has a negative effect on eosinophilia, which parallels its ability to interact with hemopoietic growth factors, such as GM-CSF [22] and G-CSF [24]. Importantly, several observations (Figures 1 and 2) support the view that the suppressive actions of IL-17A are due to activation of the iNOS-CD95 proapoptotic pathway which suppresses eosinopoiesis both in vitro [32] and in vivo [33, 35]. This would make IL-17A the first cytokine activator of this pathway to be identified.

Our study utilized extensively liquid bone-marrow culture, because it allows us to (a) concentrate on influences directly affecting the eosinopoietic precursors and their immediate environment; (b) analyse the mechanisms through which IL-17A suppresses eosinopoiesis by simple addition of a number of chemically defined factors to cultured bone-marrow of different genetic backgrounds; (c) establish dose-response relationships for cellular effects with precision; (d) define the proper timing of IL-17A introduction in the system, relative to IL-5; (e) accurately define the time-course of its effects; (f) establish the role of caspases in these effects; (g) evaluate the impact of progenitor expansion before eosinopoiesis, which requires two-step cultures.

All of the preceding studies would be very hard to do in vivo, due to the large amount of expensive reagents and number of animals necessary to test these different variables in separate experiments. In addition, this approach would be very difficult to control for effects of any or all of these ligands on targets outside the bone-marrow, which probably are relevant to prior descriptions of IL-17-expanded granulopoiesis in vivo [21, 22, 24]. By contrast, these issues were successfully addressed with cultured bone-marrow, as the liquid culture system allows us to exclude factors extrinsic to the bone-marrow and helps us gain insight of the primary consequences of IL-17A activation of IL-17RA in developing eosinophils. Indeed, our choice of bone-marrow culture as model system was rewarded by direct evidence for functional IL-17 receptors in the hemopoietic compartment, raising a number of interesting questions, which are discussed below.

The complementary use of semisolid culture provided important information. Contrary to our expectations, which were based on the results of liquid culture, IL-17A did not suppress any of the GM-CSF-stimulated colony-forming cell types in semisolid bone-marrow cultures. It did, however,* significantly increase* GM colony formation at the highest concentration tested. These findings represent a major difference relative to PGE_2_ and to a variety of cAMP-elevating agents, which are all soluble activators of the iNOS-CD95 pathway [37], and were previously shown to* inhibit*, rather than stimulate, GM-CSF-stimulated colony formation by murine bone-marrow [42].

It is important that these distinct classes of suppressive agonists have very different ways of activating their target cells. While PGE_2_ binds to widely expressed EP receptors and cAMP-elevating or mimetic agents act on cells independently of surface receptors [37], IL-17A acts through surface receptors distinct from EP. Our observation that bone-marrow lacking IL-17RA was unresponsive to IL-17A (Figure 3(d)) is consistent with current understanding of IL-17 receptor operation, since IL-17A binds directly to IL-17RA (but not to IL-17RC [7, 45]), before the resulting complex recruits IL-17RC to make a functional receptor.

We observed another difference between IL-17A and other soluble activators of this pathway, namely, its iNOS-independent effects at higher concentrations (Figure 2). While the iNOS-CD95-dependent mechanism proved more sensitive, the iNOS-independent effect is interesting in its own right, especially in view of the evidence of synergism between an NO-releasing chemical and IL-17A in bone-marrow culture from iNOS-KO mice, when both agonists are present at concentrations insufficient to have an effect. Our interpretation is that an iNOS-independent mechanism is demonstrable even under 10 ng/mL, provided that NO is present at low concentration. In other words, a little NO unveils an iNOS-independent effect of IL-17, which synergizes with NO.

On the other hand, multiple lipid (LTD4, LTC4) and cytokine (IL-13, eotaxin) mediators which share a signaling mechanism dependent on the CysLT1R block the effects of IL-17A. This highlights the importance of CysLT1R signaling in the control of eosinopoiesis [34, 36]. It further supports the concept that leukotrienes, produced in vivo after allergen challenge, account for the increase in bone-marrow eosinophil numbers and responsiveness to IL-5, which is prevented by 5-LO inactivation [35] as well as by DEC treatment [33].

Although IL-4 is not known to use CysLT1R to transduce its signals, it proved a potent antagonist of IL-17 actions at relatively low concentration (Figure 5). Furthermore, bone-marrow lacking IL-4 was exquisitely sensitive to the inhibitory effects of IL-17A, showing that IL-4 acts in vivo to decrease the responsiveness of bone-marrow cells to IL-17A. The effect of IL-4 is consistent with the observation that IL-13, another central TH2 cytokine, was also antagonic to IL-17A in these conditions. IL-4, IL-13, eotaxin, and CysLT play very important roles in allergy, and their concordant actions in bone-marrow suggest that IL-17A effects on bone-marrow are generally attenuated by mediators of allergy, in a way consistent with in vivo observations [29, 30]. One important aspect of our study is that it is concerned with naive, not sensitized, animals. Hence, the antagonism we described does not arise in a context of allergic inflammation and is likely to reflect basic relationships between IL-17A and multiple proallergic mediators. Contrary to our expectations, IL-4 at higher concentrations also suppressed eosinopoiesis, independently of the presence of IL-17A. We did not explore this effect further, partly because of the focus on IL-17 antagonism and partly because of the fact that the effect may reflect supraphysiological concentrations.

Most importantly, the interactions of IL-17 (dependent on iNOS and CD95 for its effectiveness) with LTD4, IL-13, and eotaxin (all dependent on the 5-LO pathway for their antagonistic effects) provide evidence that the iNOS-CD95L pathway is articulated with the 5-LO pathway via CysLT1R. Ultimately, this supports the view that the iNOS/5-LO enzyme duo is central to regulation of eosinopoiesis [35], highlighting the need of better understanding of their expression, activity, and interactions in bone-marrow.
